# Effects of Combined Oregano Essential Oil and *Macleaya cordata* Extract on Growth, Antioxidant Capacity, Immune Function, and Fecal Microbiota in Broilers

**DOI:** 10.3390/vetsci12121206

**Published:** 2025-12-16

**Authors:** Yi Lu, Zhenyue Li, Zitong Yang, Ran Zhu, Mengxi Yan, Zhuhua Liu, Mingli Liu, Yuan Wang, Jue Wang, Qi Wang, Juxiang Liu, Cheng Zhang, Xuejing Wang, Huan Cui

**Affiliations:** 1 College of Veterinary Medicine, Hebei Agricultural University, Baoding 071000, China; 2 College of Medicine, Yanbian University, Yanji 133002, China; 3 State Key Laboratory of Animal Nutrition and Feeding, Institute of Animal Sciences, Chinese Academy of Agricultural Sciences, Beijing 100193, China; 4 The Animal Husbandry and Veterinary Institute of Hebei, Baoding 071001, China

**Keywords:** phytogenic feed additive, gut microbial ecology, 16S rRNA gene sequencing, antibiotic alternative, poultry production

## Abstract

With the growing demand for antibiotic-free and sustainable poultry production, plant-derived antimicrobials have emerged as promising alternatives. However, a systematic understanding of the combined effects of oregano essential oil (OEO) and *Macleaya cordata* extract (MCE) on the broiler gut microbiome remains lacking. This study employed an integrated “structure–function–phenotype” framework to investigate the individual and combined (OMS) effects of OEO and MCE on gut microecological remodeling and its coupling with host growth, metabolic, and immune phenotypes. A total of 960 one-day-old broiler chicks were individually weighed and then randomly allocated to four treatments using body-weight-stratified randomization, with 6 replicate pens per treatment and 40 birds per pen, to ensure similar initial body weight across groups. Over a 42-day trial, we evaluated growth performance, serum biochemistry, antioxidant status, and immune parameters. Compared to the control, the OMS treatment significantly enhanced average daily feed intake (ADFI) and average daily gain (ADG), increased serum total protein (TP), and decreased blood urea nitrogen (BUN), triglycerides (TG), total cholesterol (TC), and alkaline phosphatase (ALP). However, the feed-to-gain ratio (F/G) was also higher in the OMS group, indicating that the improvement in growth rate did not translate into enhanced feed efficiency but was primarily driven by increased feed consumption. OMS also improved overall antioxidant capacity and key enzyme activities, elevated immunoglobulin levels, and reduced pro-inflammatory cytokines. Notably, OMS maintained *Lactobacillus* dominance, enriched *Bacteroides, Enterococcus*, and *Butyricicoccus*, and reduced *Escherichia–Shigella*. Functional predictions via PICRUSt2 suggested enhanced metabolic pathways related to antioxidant and immune functions; however, these results represent inference-based predictions and should be interpreted cautiously. Overall, the combination of OEO and MCE exerted synergistic benefits on growth, physiological health, and gut microbiota, supporting its potential as a phytogenic strategy for antibiotic-free broiler production.

## Simple Summary

Antibiotic growth promoters have been banned or strictly restricted in many countries, creating an urgent need for safe and effective alternatives to support broiler health and performance. Oregano essential oil and *Macleaya cordata* extract are two phytogenic additives with promising antimicrobial, antioxidant, and anti-inflammatory properties, but their combined effects in broilers are not fully understood. In this study, 960 broilers were randomly assigned to four treatments: a control group, a group receiving oregano essential oil (OEO), a group receiving *Macleaya cordata* extract (MCE), and a group receiving a combination of the two (OMS). The combined OMS treatment improved growth performance and feed efficiency, enhanced serum antioxidant and immune indices, and modulated fecal microbiota by enriching beneficial bacteria and reducing potentially harmful taxa. These findings suggest that the combination of OEO and MCE can be used as a practical phytogenic strategy to improve broiler productivity and gut health in antibiotic-free production systems.

## 1. Introduction

Under the global intensive poultry production system, high stocking density, environmental stress, and long-term exposure of broilers to pathogenic microorganisms have become major challenges affecting broiler health and productivity. These stressors can lead to intestinal microbiota dysbiosis, compromise the mucosal barrier, and impair immune function, thereby impairing growth performance and elevating the risk of antibiotic dependence [1,2]. Antibiotic growth promoters have traditionally improved poultry production by reducing subclinical pathogen burden, decreasing intestinal inflammation and epithelial turnover, and lowering the energy costs associated with immune activation. They may also enhance nutrient utilization by modulating gut microbial composition [2,3,4]. However, the overuse of antibiotics has contributed to the emergence of bacterial resistance, accumulation of drug residues, and environmental contamination, posing serious threats to public health and food safety [4,5]. To mitigate these challenges, the European Union fully banned the use of antibiotic growth promoters in animal feed in 2006, followed by similar regulations in the United States, Canada, and other countries [3,6,7]. Since 2020, China has implemented a comprehensive ban on antibiotics in feed, marking a significant step toward sustainable, antibiotic-free poultry production [8].

Driven by the dual impetus of the “antibiotic-free” policy and increasing consumer demand for safe animal-derived products, the development of natural plant extracts with antimicrobial, anti-inflammatory, growth-promoting, and immunomodulatory properties has become a major research focus in the field of poultry nutrition and health. Among these, oregano essential oil (OEO) and *Macleaya cordata* extract (MCE) emerged as two of the most promising antibiotic alternatives due to their potent pharmacological activities and excellent safety profiles [9,10]. OEO is a volatile essential oil derived from the Lamiaceae plant *Origanum vulgare* L., whose major active constituents are monoterpenoid phenols such as carvacrol and thymol. These bioactive compounds exhibit broad-spectrum antimicrobial activity by disrupting the structural integrity of bacterial cell membranes, inhibiting energy metabolism, and preventing biofilm formation [11,12]. Although many plant-derived bioactive compounds exhibit broad-spectrum antimicrobial activity, their efficacy is highly concentration-dependent. Recent studies have shown that OEO not only promotes intestinal microbiota balance and stimulates the proliferation of beneficial bacteria such as *Lactobacillus* but also enhances the host’s antioxidant defense by modulating redox status and activating the Nrf2 signaling pathway [13,14]. In addition, OEO has been reported to stimulate appetite, enhance digestive enzyme secretion, and improve intestinal villus morphology, thereby markedly increasing feed conversion efficiency and growth performance in broilers. *Macleaya cordata*, a member of the Papaveraceae family, is rich in various benzophenanthridine alkaloids, primarily sanguinarine and chelerythrine, both of which possess extensive biological activities, including antibacterial, anti-inflammatory, antioxidant, and immunomodulatory effects [10,15,16]. Sanguinarine has been demonstrated to alleviate intestinal inflammation by inhibiting the NF-κB signaling pathway and downregulating pro-inflammatory cytokines such as tumor necrosis factor-α (TNF-α) and interleukin-1β (IL-1β) [17]. Moreover, alkaloids from *M. cordata* have been shown to upregulate the expression of tight junction proteins (e.g., Occludin and Claudin), thereby strengthening intestinal barrier integrity and enhancing mucosal repair [18]. In addition, MCE can modulate the intestinal microbiota composition, elevate immunoglobulin levels (IgA and IgG), and improve immune homeostasis, exhibiting an outstanding “antioxidation–immunity–barrier” synergistic effect [10,15].

In recent years, the combined application of plant-derived bioactive compounds has emerged as an important strategy to enhance the efficacy of antibiotic alternatives. Studies have demonstrated that different plant extracts can exert complementary effects due to their diverse chemical structures and mechanisms of action, resulting in synergistic enhancement through the coordinated regulation of antioxidant and anti-inflammatory pathways, as well as cooperative remodeling of the gut microbial community [19,20,21]. Our previous research demonstrated that the combined supplementation of OEO and MCE significantly improved broiler growth performance, enhanced intestinal antioxidant capacity, and strengthened immune responses [22]. However, systematic investigations into how OEO and MCE—individually and especially in combination—reshape the structural composition and functional metabolic potential of the intestinal microbiota remain limited. Specifically, the field lacks comprehensive studies characterizing (i) microbiota community shifts under combined phytogenic supplementation, (ii) alterations in microbiota-derived functional pathways related to nutrient utilization and host physiology, and (iii) the microbiota-mediated mechanisms through which OEO and MCE together influence metabolic homeostasis and immune regulation. These gaps highlight the need to explore how the co-administration of OEO and MCE modulates gut microecology and its coupling with host phenotypes.

Therefore, this study systematically evaluated the effects of the combined use of OEO and MCE on growth performance, serum biochemical parameters, antioxidant and immune functions, as well as the structural and functional profiles of the intestinal microbiota in broilers. By integrating 16S rRNA high-throughput sequencing with functional prediction analyses, this work aimed to elucidate the molecular and microecological mechanisms underlying the synergistic effects of OEO and MCE in improving gut health and production performance. The findings are expected to provide a theoretical basis for the rational formulation and precise application of plant-derived antibiotic alternatives, as well as offer new technical and scientific support for achieving antibiotic-free poultry production and promoting the sustainable development of the poultry industry.

## 2. Materials and Methods

### 2.1. Animal Experimental Design and Treatments

#### Animal Grouping

All experimental procedures were approved by the Animal Ethics Committee of Hebei Agricultural University (document number of approval: 20240709) and were conducted in accordance with the relevant guidelines and regulations for the care and use of experimental animals. A total of 960 one-day-old healthy Hubbard broiler chicks with uniform body weight were obtained from Enkang Poultry Co., Ltd. (Baoding, China). Each treatment comprised six replicates with 40 birds per replicate. Broilers were reared in cages throughout the experimental period. Each cage had a floor area of 2.25 m^2^ and housed 5 birds, corresponding to a stocking density of approximately 2.22 birds/m^2^. The basal diet was formulated according to the nutritional requirements for broilers [23], with its composition and nutrient levels shown in Supplementary Table S1. The experiment followed a completely randomized design, with birds randomly allocated into four treatment groups: Control group (Control), OEO oral solution group (OEO), MCE oral solution group (MCE), and the combined oral solution group of OEO and MCE (OMS). The ambient temperature was maintained at 22–25 °C, and the relative humidity was controlled at 50–60%. Lighting and ventilation conditions complied with standard poultry management requirements [24,25]. Feed was automatically provided twice daily (at 06:00 and 15:00) using a fully automated feeding system, and birds had ad libitum access to feed and water throughout the experimental period. All birds received the same basal diet, while oral solutions were administered via the drinking water system to differentiate treatments. The preparation of OEO and MCE oral solutions followed the procedure described in our previous study [22]. Treatment details were as follows: Control group—pure water; OEO group—drinking water supplemented with OEO oral solution; MCE group—drinking water supplemented with MCE oral solution; OMS group—drinking water supplemented with a mixture of OEO and MCE oral solution (5% OEO, 1% MCE, 25% polyoxyethylene (40) hydrogenated castor oil, 0.02% antioxidant 2,6-di-tert-butyl-p-cresol, and water up to 100%). In the OMS formulation, the concentrations of the major active constituents were 42.59 mg/mL of carvacrol and 6.51 mg/mL of sanguinarine. Based on the administration rate (125 mL per 1000 L of drinking water), the final concentrations in the drinking water were 5.32 mg/L of carvacrol and 0.81 mg/L of sanguinarine. All solutions were delivered once daily via an automated watering system and continuously administered until the end of the trial. All solutions were thoroughly mixed before use to ensure uniform dispersion and concentration stability.

During the experimental period, the health status, feeding behavior, and mortality of broilers were monitored daily, and any abnormal individuals were promptly recorded. Feed intake and feed residues were measured daily for each replicate to calculate the average daily feed intake (ADFI). On day 42, all broilers were weighed to determine the average daily gain (ADG) and feed-to-gain ratio (F/G), following the calculation methods described previously [26]. Samples were collected at a standardized postprandial time point rather than after fasting. At the end of the experiment, all broilers (*n* = 960 birds in total, 240 birds per treatment) were first sampled for peripheral blood via wing vein puncture and were subsequently euthanized by carbon dioxide inhalation in accordance with the approved animal welfare protocol. Approximately 5 mL of blood was drawn from each broiler into sterile centrifuge tubes. After clotting at room temperature, samples were centrifuged at 2000× *g* for 15 min at 4 °C. Serum samples were visually inspected for hemolysis immediately after centrifugation. Samples exhibiting any degree of discoloration indicative of hemolysis were excluded and recollected to ensure the accuracy of biochemical and immunological analyses. Serum was aliquoted into sterile microtubes and stored at −80 °C for subsequent analyses.

### 2.2. Major Reagents

All major biochemical and immunological reagents used in this study were commercially available and prepared according to the manufacturers’ instructions before use. Kits for serum biochemical analyses included total protein (TP, A045-2-2), albumin (ALB, A028-1-1), blood urea nitrogen (BUN, C013-2-1), triglyceride (TG, A110-2-1), total cholesterol (TC, A111-2-1), and alkaline phosphatase (ALP, A059-2-1), all obtained from Nanjing Jiancheng Bioengineering Institute (Nanjing, China). Antioxidant-related assays included total antioxidant capacity (T-AOC, A015-1-1), glutathione peroxidase (GSH-Px, A005-1-2), malondialdehyde (MDA, A003-1-2), and superoxide dismutase (SOD, A001-3-2), also purchased from Nanjing Jiancheng Bioengineering Institute. Immune-related parameters were determined using enzyme-linked immunosorbent assay (ELISA) kits. Chicken immunoglobulin A (IgA) and immunoglobulin M (IgM) ELISA kits were obtained from Abcam (Cambridge, UK), while the chicken IgG ELISA kit (DL-IGG-CH) was supplied by Donglin Technology Co., Ltd. (Beijing, China). ELISA kits for chicken interferon-α (IFN-α, ECH0024), interleukin-4 (IL-4, ECH0044), and interleukin-6 (IL-6, ECH0046) were purchased from Fine Biotech Co., Ltd. (Nanjing, China). Plant extracts were sourced as follows: OEO (carvacrol content 45 mg/mL, Cat. No. O137777) from Aladdin Biochemical Technology Co., Ltd. (Shanghai, China) and MCE (sanguinarine content 6.5 mg/mL, Cat. No. MC70) from Hanqing Biotechnology Co., Ltd. (Huaihua, China). All plant extracts were filtered through 0.22 μm membranes for sterilization and stored at 4 °C in the dark before use. For intestinal samples, DNA was extracted using a commercial fecal DNA extraction kit (DP328, Tiangen Biotech Co., Ltd., Beijing, China). PCR products were purified with the AxyPrep DNA Gel Extraction Kit (Axygen, Union City, CA, USA).

### 2.3. Fecal Microbiota Analysis

Fecal samples were analyzed by Megagen Biotechnology Co., Ltd. (Guangdong, China) using high-throughput 16S rRNA gene sequencing. The complete sequencing dataset has been deposited in the NCBI GenBank repository under the BioProject accession number PRJNA1354574. Total genomic DNA was extracted using a commercial fecal DNA extraction kit. DNA concentration was measured with a NanoDrop spectrophotometer (Thermo Scientific, Wilmington, DE, USA), and DNA integrity was evaluated by electrophoresis using the FlashGel system (Lonza, Rockland, ME, USA) to ensure purity and integrity suitable for downstream amplification. The V3–V4 hypervariable regions of the bacterial 16S rRNA gene were amplified using universal primers, and PCR conditions were conducted as previously described [27]. The V3–V4 region of the bacterial 16S rRNA gene was amplified using primers 341F (5′-CCTACGGGNGGCWGCAG-3′) and 806R (5′-GGACTACHVGGGTATCTAAT-3′). PCR reactions were performed in a 25 μL mixture containing 12.5 μL of 2× KAPA HiFi HotStart ReadyMix, 0.2 μM of each primer, and approximately 10 ng of template DNA. Thermal cycling conditions were as follows: initial denaturation at 95 °C for 3 min, followed by 25 cycles of denaturation at 95 °C for 30 s, annealing at 55 °C for 30 s, and extension at 72 °C for 30 s, with a final extension at 72 °C for 5 min. PCR products were verified by 1.5% agarose gel electrophoresis. Purified amplicons were sequenced on the Illumina MiSeq platform (Illumina, San Diego, CA, USA). Fecal 16S rRNA gene sequencing data were processed using QIIME 2 (version 2025.4). Paired-end reads were first demultiplexed by the sequencing provider and subjected to quality filtering to remove low-quality bases and residual adapter/primer sequences. Chimeric sequences were identified and removed. The high-quality reads were then clustered into operational taxonomic units (OTUs) at 97% sequence similarity, which were used as features for downstream diversity analyses. Taxonomic assignment of representative OTU sequences was performed against the SILVA reference database. The resulting OTU table and representative sequences were subsequently imported into PICRUSt2 (version 2.6.2) to generate predicted functional profiles based on 16S rRNA gene data. Alpha diversity indices, including Chao1 and Shannon indices, were calculated using QIIME to assess species richness and diversity. Beta diversity was evaluated using Bray–Curtis distances, followed by principal coordinates analysis (PCoA) and hierarchical clustering to compare microbial community composition among different treatment groups. Beta-diversity differences among treatments were evaluated using Bray–Curtis dissimilarity matrices and tested statistically by PERMANOVA (permutational multivariate analysis of variance) with 999 permutations, implemented in QIIME 2. Additionally, PICRUSt2 software was employed to predict the potential metabolic functions of the microbial communities, and KEGG (Kyoto Encyclopedia of Genes and Genomes) pathway annotation was performed to functionally evaluate the effects of OEO and MCE supplementation on the intestinal microbiota. PICRUSt2 functional predictions were evaluated using the Nearest Sequenced Taxon Index (NSTI) to determine inference reliability. The average NSTI score across samples was 0.06, which is within the acceptable range for poultry gut microbiome datasets and indicates high confidence in functional prediction.

### 2.4. Statistical Analysis

All data were analyzed using SPSS v19.0 (IBM Corp., Armonk, NY, USA). Results are presented as mean ± standard deviation (Mean ± SD). Before applying one-way analysis of variance (ANOVA), the data for each variable were tested for normality using the Shapiro–Wilk test and for homogeneity of variances using Levene’s test. When necessary, data were transformed (e.g., log10 transformation for positively skewed variables and arcsine square-root transformation for percentage or proportion data) to better meet these assumptions. If the transformed data still did not satisfy normality and/or homoscedasticity, the Kruskal–Wallis non-parametric test was used instead of ANOVA, followed by Dunn–Bonferroni post hoc comparisons. For variables that met the assumptions, differences among treatment groups were evaluated by one-way ANOVA, followed by Duncan’s multiple range test for pairwise comparisons when a significant main effect was detected. Statistical significance was defined as *p* < 0.05, and *p* ≥ 0.05 was considered not significant.

## 3. Results

### 3.1. Growth Performance

As summarized in Table 1, the combined supplementation of OEO and MCE (OMS group) yielded the most pronounced benefits on broiler growth performance. Specifically, the OMS group exhibited significant increases in both ADFI and ADG compared to the Control (*p* < 0.05). In contrast, the OEO and MCE groups, when administered individually, significantly improved ADG but not ADFI relative to the Control. Notably, F/G was significantly higher in the OMS group than in all other groups (*p* < 0.05). However, this increase in F/G occurred alongside a substantial boost in overall weight gain, confirming a potent net growth-promoting effect. Together, these findings suggest that the efficacy of OEO and MCE in promoting broiler growth is synergistically enhanced when they are co-administered.

### 3.2. Effects of OMS on Serum Biochemical Parameters in Broilers

The effects of OEO and MCE on serum biochemical parameters are presented in Table 2. The combined OMS treatment significantly enhanced serum TP and reduced BUN, TG, TC, and ALP activity compared to the Control (*p* < 0.05). No significant change was observed in ALB levels across groups. Notably, the OMS group demonstrated significantly greater reductions in TG than all other groups and in TC than the Control and MCE groups (*p* < 0.05). These findings indicate that the OMS combination synergistically improves protein and lipid metabolism. The decrease in ALP may indicate a trend toward improved hepatic metabolic status, as lower circulating ALP activity has been associated with reduced hepatocellular stress in poultry [28,29]. However, this interpretation should be made cautiously given that ALP is influenced by multiple physiological factors. Collectively, the results underscore the role of OMS in promoting systemic metabolic homeostasis in broilers.

### 3.3. Effects of OMS on Antioxidant and Immune Parameters in Broilers

As shown in Table 3, different treatments had significant effects on serum antioxidant and immune-related parameters in broilers. Regarding antioxidant indices, T-AOC was significantly higher in the OMS group than in the Control and MCE groups (*p* < 0.05), while the OEO group also showed a significant increase compared with the Control (*p* < 0.05). GSH-Px activity was highest in the OMS group, significantly exceeding that of all other groups (*p* < 0.05); in addition, GSH-Px activity in the MCE and OEO groups was significantly higher than that in the Control. SOD activity was significantly elevated in the OMS group compared with the Control (*p* < 0.05), whereas MDA levels did not differ significantly among groups (*p* > 0.05). Regarding immune indices, serum IgA, IgM, and IgG levels in the OMS group were significantly higher than those in the Control (*p* < 0.05), indicating a marked enhancement of humoral immunity. At the same time, pro-inflammatory cytokines, including TNF-α, IL-1β, IL-4, and IL-6, were significantly lower in the OMS group than in the Control (*p* < 0.05), suggesting effective suppression of inflammatory responses. Collectively, these results indicate that OMS group significantly improved antioxidant status and immune function in broilers by enhancing antioxidant enzyme activities, increasing immunoglobulin levels, and downregulating pro-inflammatory cytokine expression. This suggests a potential “antioxidant–immune–inflammatory” synergistic regulatory mechanism contributing to overall health homeostasis.

### 3.4. OTU Statistics and α-Diversity Analysis

**Figure 1:**
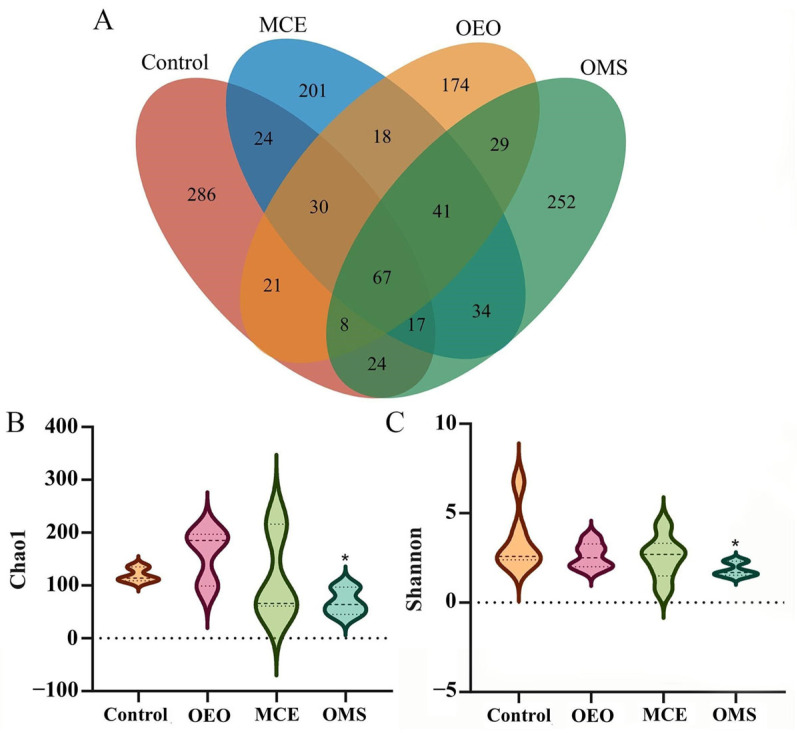
OTU statistics and α-Diversity analysis in different experimental groups. (**A**) Venn diagram showing shared and unique OTUs among groups. (**B**) α-Diversity analysis based on the Chao1 index. (**C**) α-Diversity analysis based on the Shannon index. * *p* < 0.05.

On day 42 of the experiment, fecal samples were collected from healthy broilers in each treatment group, with six samples per group, resulting in a total of 24 samples for 16S rRNA gene sequencing analysis. After applying low-abundance filtering, the Control group harbored 286 unique OTUs, while the OEO, MCE, and OMS groups contained 174, 201, and 252 unique OTUs, respectively. A total of 67 core OTUs were shared among all four groups, indicating that these taxa represent a relatively stable microbial consortium under different feeding conditions. In addition, several OTUs were shared among treatment groups, such as 34 OTUs shared between the MCE and OMS groups and 29 OTUs shared between the OEO and OMS groups, suggesting partial overlap and complementary effects in modulating gut microbial community structure among the different plant extract treatments. Alpha diversity indices (Chao1 and Shannon) were used to evaluate species richness and diversity. Violin plots (Figure 1B,C) illustrate the distribution of these indices across treatment groups. Compared with the Control, the OMS group exhibited a slight decrease in both Chao1 and Shannon indices, indicating that the combined application of OEO and MCE moderately reduced intestinal microbiota richness and evenness. This change may be associated with the enrichment of dominant beneficial taxa, such as *Lactobacillus* and *Bacteroides*, and a relative reduction in rare or opportunistic taxa, thereby restructuring the gut microbiota toward a more stable and functionally optimized state.

### 3.5. β-Diversity Analysis of the Microbiota

**Figure 2:**
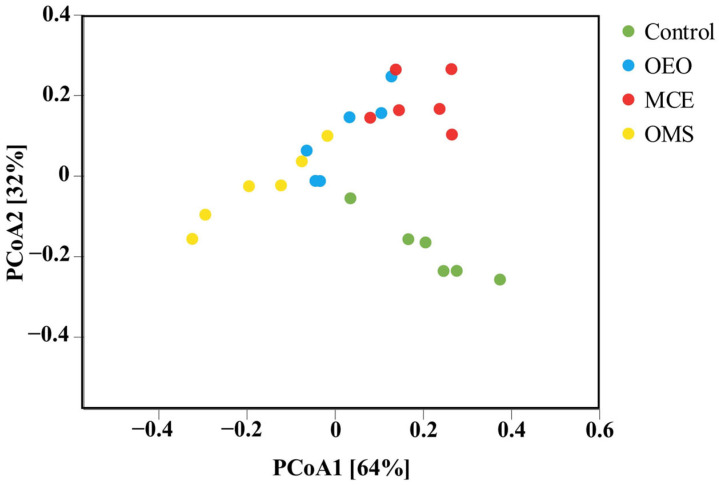
Principal coordinate analysis (PCoA) of gut microbial communities based on the Bray–Curtis distance.

To further investigate the similarities and differences in gut microbial community structure among different treatment groups, principal coordinate analysis (PCoA) based on Bray–Curtis distances was performed to evaluate community composition dissimilarities (Figure 2). Each point in the plot represents an individual sample, and points of the same color correspond to the same treatment group. The spatial distance between points reflects the similarity of microbial communities—closer points indicate more similar community structures, whereas greater distances indicate larger differences. The results showed that the Control group was clearly separated from the OEO, MCE, and OMS groups in the PCoA plot, indicating substantial shifts in microbial community structure following supplementation. This visual separation was statistically supported by a PERMANOVA test, which revealed significant differences in β-diversity among treatments (*p* < 0.05). In contrast, the OEO, MCE, and OMS groups exhibited partial overlap, suggesting some similarity in their microbial composition. Notably, samples from the OMS and MCE groups clustered more closely together, indicating relatively small differences in community structure and higher ecological similarity. Overall, both individual and combined supplementation of OEO and MCE significantly altered the overall composition of the broiler gut microbiota, leading to distinct β-diversity patterns between the treatment groups and the Control. Furthermore, the OMS group exhibited a more concentrated and stable community distribution, suggesting a synergistic effect in reshaping gut microbial structure and optimizing community stability.

### 3.6. Genus-Level Composition of the Gut Microbiota at the Same Time Point

**Figure 3:**
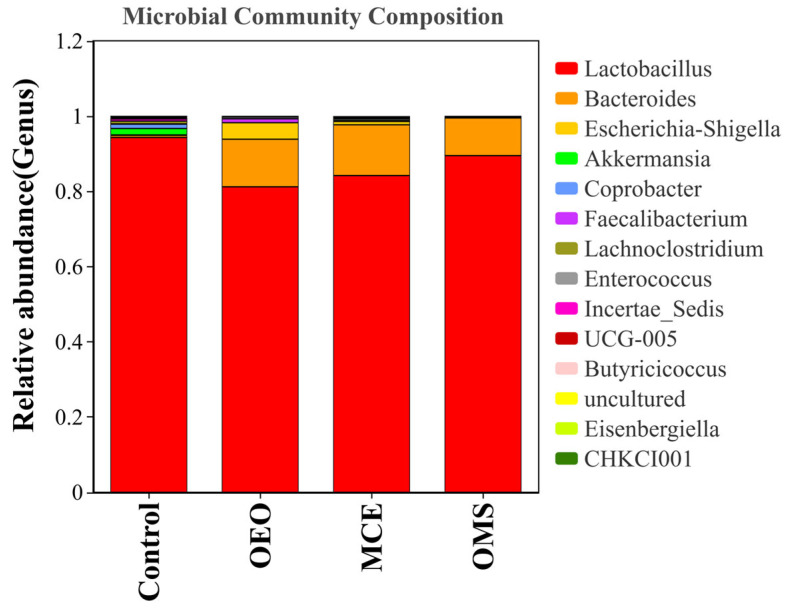
Relative abundance of gut microbiota at the genus level.

The genus-level composition of the gut microbiota was analyzed to elucidate structural changes induced by the treatments (Figure 3). *Lactobacillus* and *Bacteroides* were identified as the dominant genera. While *Lactobacillus* remained predominant across all groups, its relative abundance decreased in the OEO (81.20%) and MCE (84.19%) groups compared to the Control (94.31%), but it was better maintained in the OMS group (89.49%). Conversely, the abundance of *Bacteroides* increased in all treatment groups. A key finding pertained to the opportunistic pathogen *Escherichia–Shigella*. Its abundance markedly increased to 4.39% in the OEO group and 0.87% in the MCE group, relative to the Control (0.12%). In stark contrast, the OMS group effectively suppressed *Escherichia–Shigella* (0.07%). These results demonstrate that while individual OEO or MCE supplementation modulated microbiota structure, it was insufficient to control potential pathogens. In contrast, the OMS combination synergistically maintained core beneficial bacteria and potently inhibited opportunistic pathogens, thereby promoting a more stable and homeostatic gut microbial environment.

### 3.7. Genus-Level Abundance Clustering Analysis of Gut Microbiota at the Same Time Point

**Figure 4:**
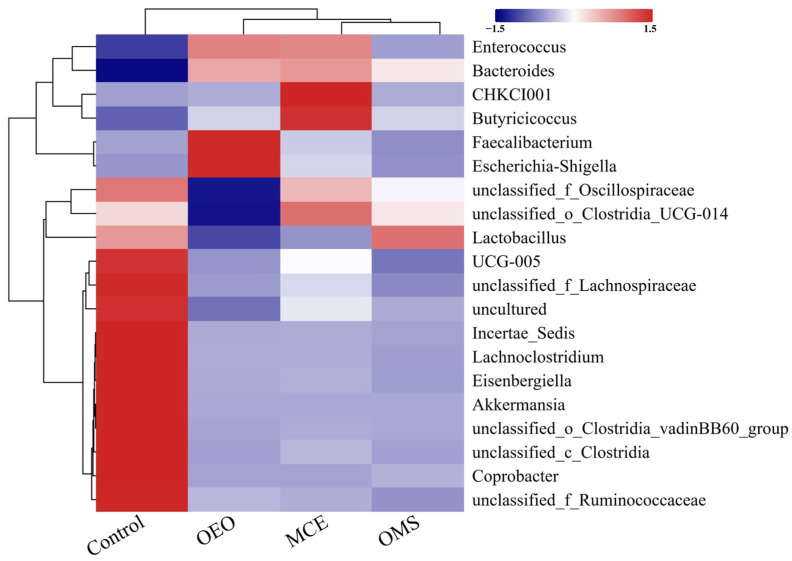
Correlation heatmap of the Top 20 genera. Red and blue colors represent positive and negative correlations, respectively.

To further investigate the structural characteristics and dynamic changes in the broiler gut microbiota, genus-level comparisons were performed among treatment groups. Based on 16S rRNA sequencing results, genera with relative abundances greater than 0.01% were selected, and the top 20 most abundant genera (Top 20) were used to generate a bar chart illustrating differences in community composition among groups (Figure 3). The results indicated that the dominant genera in the broiler gut microbiota were *Lactobacillus* and *Bacteroides*, and their relative abundances largely reflected the stability and health of the intestinal microecosystem. At day 42, the relative abundances of these two genera were as follows: Control group, 94.31% and 0.51%; OEO group, 81.2% and 12.51%; MCE group, 84.19% and 13.15%; OMS group, 89.49% and 9.84%. *Lactobacillus* remained the predominant genus across all groups, while the relative abundance of *Bacteroides* was significantly higher in the OEO, MCE, and OMS groups compared with the Control. Notably, the decrease in *Lactobacillus* abundance was minimal in the OMS group, suggesting that the combined treatment helped maintain the stability of core beneficial bacteria. In addition, the opportunistic pathogenic genus *Escherichia–Shigella* showed significant intergroup differences. Its relative abundance in the Control, OEO, MCE, and OMS groups was 0.12%, 4.39%, 0.87%, and 0.07%, respectively. The increase in this genus in the OEO and MCE groups indicates that single plant extract supplementation may have limited inhibitory effects on potential pathogens, whereas the OMS group exhibited a marked decrease, suggesting a stronger synergistic antibacterial effect of the combined treatment. Overall, these findings demonstrate that the combined application of OEO and MCE not only promotes the stability of dominant beneficial genera (such as *Lactobacillus* and *Bacteroides*) but also effectively suppresses opportunistic pathogens (such as *Escherichia–Shigella*), thereby contributing to the maintenance of gut microbial homeostasis and host health (Figure 4). This result further confirms the synergistic modulatory effect between OEO and MCE and provides experimental evidence for the microecological mechanism of plant-based composite antibiotic alternatives.

### 3.8. KEGG Functional Analysis

**Figure 5:**
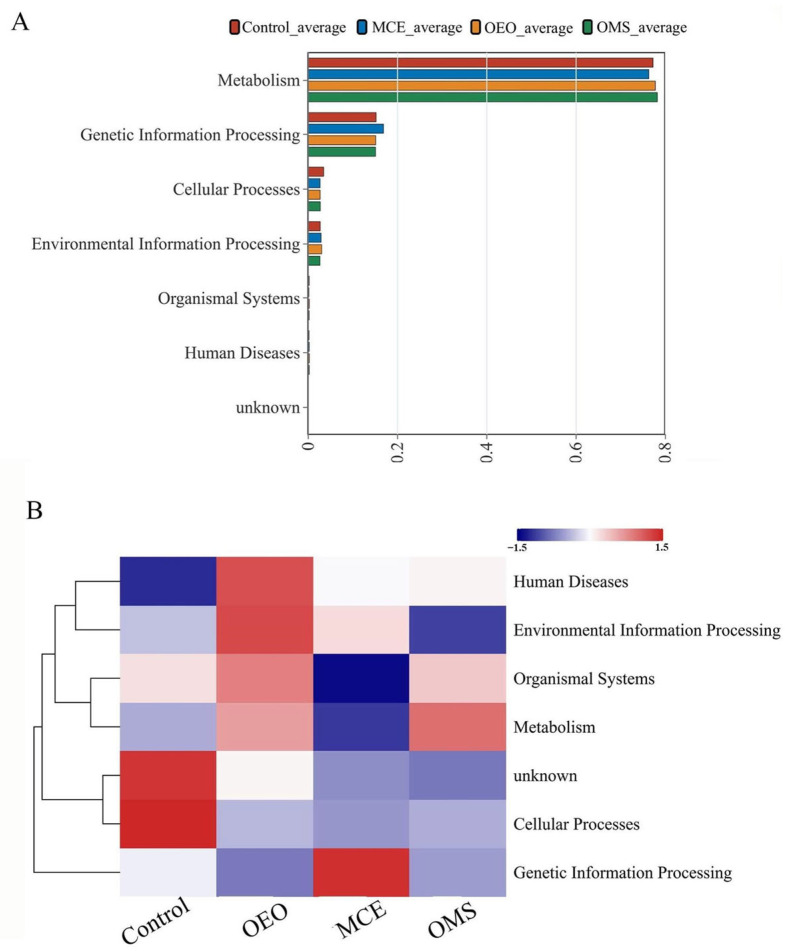
KEGG functional analysis of gut microbiota in different treatment groups. (**A**) Pathway composition analysis. (**B**) Pathway clustering analysis.

Based on putative KEGG functional annotations inferred from PICRUSt2, we compared the predicted metabolic potentials of the gut microbiota among treatment groups (Figure 5). As shown in Figure 5A, all groups were mainly enriched in metabolism-related pathways, a pattern commonly observed in poultry gut microbiota. These patterns likely reflect the general metabolic roles of commensal communities in nutrient processing and energy turnover. When comparing treatment groups (Figure 5B), the OMS group showed higher predicted relative abundances in several metabolism-associated categories, including carbohydrate and amino acid metabolism and pathways linked to short-chain fatty acid (SCFA) biosynthesis. Pathways related to genetic information processing and cellular functions also showed modest increases. Conversely, the Control group displayed relatively higher representation of environmental information processing pathways. Given that these findings are derived from 16S rRNA-based predictive modeling, they represent putative functional shifts rather than direct measurements of microbial activity. Nonetheless, the predicted profiles suggest that OMS supplementation may favor microbiota configurations with enhanced metabolic capacity, which could contribute to improved physiological outcomes observed in the host.

## 4. Discussion

This study provides a comprehensive evaluation of the synergistic effects elicited by the combined application of OEO and MCE in broilers. Our findings demonstrate that OMS acts through a multi-level mechanism, integrating enhancements in nutrient metabolism, antioxidant capacity, immune regulation, and gut microbiota remodeling, which collectively underpin the observed improvements in growth performance and overall health.

### 4.1. Synergistic Enhancement of Growth and Metabolic Health

The superior growth performance (ADFI, ADG) in the OMS group underscores a synergistic interaction between OEO and MCE. This can be attributed to their complementary roles in promoting gastrointestinal function. OEO bioactive compounds, carvacrol and thymol, are known to stimulate digestive secretions and enhance gut motility [30,31,32], while alkaloids in MCE, such as sanguinarine, strengthen intestinal barrier integrity and nutrient absorption by upregulating tight junction proteins [33]. This synergy was further reflected in serum biochemical parameters. The elevated TP and reduced BUN suggest enhanced protein synthesis and utilization. Concurrently, the lowered TG, TC, and ALP indicate improved lipid metabolism and reduced hepatic metabolic burden, potentially through the modulation of key enzymes like ACC, FAS, and CPT-1 [34]. The decrease in ALP, in particular, points to alleviated cellular stress, likely resulting from mitigated intestinal inflammation and endotoxin translocation. Although the OMS group exhibited a higher feed-to-gain ratio (F/G), this increase should be interpreted in the context of the markedly enhanced growth dynamics. OMS significantly elevated ADFI, and this increased nutrient intake supported a greater ADG; however, because the proportional increase in ADFI slightly exceeded the increase in ADG, the statistical outcome appeared as a higher F/G. This does not indicate reduced efficiency; rather, it reflects a voluntary rise in feed consumption driven by improved metabolic capacity and physiological status. The absence of adverse changes in serum metabolites or inflammatory markers further suggests that the increased feed intake represents healthy metabolic upregulation rather than inefficiency or nutrient wastage. In addition to broilers, the benefits of phytogenic feed additives have also been demonstrated in other poultry species. For example, dietary supplementation with garlic powder was shown to significantly improve growth performance, carcass traits, and meat quality in Japanese quails, further supporting the notion that plant-derived compounds can function as effective natural growth promoters across diverse avian models [35]. Collectively, such findings highlight the broader relevance of phytogenic alternatives as the poultry industry increasingly moves toward reducing or eliminating antibiotic growth promoters.

### 4.2. Integrated Activation of Antioxidant and Immune Defenses

The OMS group exhibited a robust activation of the systemic antioxidant defense system, as evidenced by significant increases in T-AOC, GSH-Px, and SOD activities. This effect is mechanistically grounded: carvacrol in OEO can activate the Nrf2/HO-1 pathway to upregulate antioxidant enzymes [36], while sanguinarine in MCE suppresses ROMS generation by inhibiting NADPH oxidase. Their combined action creates a powerful redox-balancing effect. Although MDA levels did not differ significantly among treatments, the marked increases in SOD, GSH-Px, and T-AOC suggest that OMS primarily enhanced the antioxidant defense system rather than producing immediate measurable reductions in lipid peroxidation. This indicates an improved capacity to counteract oxidative stress, even if downstream markers such as MDA had not yet changed at the time of sampling.

This enhanced antioxidant capacity was coupled with a marked immunomodulatory shift. The elevated levels of immunoglobulins (IgA, IgM, IgG) and the suppression of pro-inflammatory cytokines (TNF-α, IL-1β, IL-4, IL-6) indicate a dual action of strengthening humoral immunity while curbing excessive inflammation. At the same time, pro-inflammatory cytokines, including TNF-α, IL-1β, IL-4, and IL-6, were significantly lower in the OMS group compared with the Control (*p* < 0.05). Importantly, all cytokine concentrations remained within the physiological ranges reported for healthy broilers, suggesting that the observed reductions reflect a normalization of inflammatory tone rather than immune suppression [37]. The rise in IgA is critical for reinforcing the gut mucosal barrier. The anti-inflammatory effect can be linked to sanguinarine’s inhibition of the NF-κB and MAPK pathways [38], complemented by OEO’s ability to mitigate inflammation secondary to oxidative stress.

### 4.3. Remodeling of a Health-Promoting Gut Microbiota

Our data indicate that the gut microbiota is an important target of the OMS treatment. Although alpha diversity indices showed a slight reduction in the OMS group, such changes should be interpreted with caution, as lower diversity does not necessarily imply impaired ecological function. In many host-associated microbial systems, community composition and stability can be more relevant to physiological outcomes than diversity alone [39]. The clear separation among groups in the PCoA plot suggests that OMS supplementation reshaped the gut microbial community structure (β-diversity), but the functional implications of this restructuring require further validation through metagenomic or metabolomic approaches.

At the genus level, OMS promoted a “core beneficial microbiota stabilization and pathogen suppression” profile. It maintained a high abundance of *Lactobacillus* and enriched for beneficial genera like *Bacteroides* (polysaccharide degradation, anti-inflammation), *Enterococcus* (bacteriocin production, immune stimulation), and the butyrate producer *Butyricicoccus* (barrier integrity, immune balance) [40,41,42,43,44,45,46]. Crucially, OMS potently suppressed the opportunistic pathogen *Escherichia–Shigella*, a feat not achieved by either extract alone, highlighting their synergistic antimicrobial action via membrane disruption (OEO phenolics) and metabolic interference (MCE alkaloids). The increase in *Escherichia–Shigella* in the OEO group (4.39%) was unexpected. This pattern may reflect selective pressure imposed by oregano phenolics, which can suppress some competing taxa while allowing more tolerant *Escherichia–Shigella* strains to persist. Notably, this increase did not occur in the OMS group, where *Escherichia–Shigella* was markedly reduced, suggesting that the inclusion of MCE may counteract the unintended enrichment seen with OEO alone. Based on 16S rRNA profiling, the combined supplementation was associated with shifts in fecal community composition, including an apparent enrichment of commensal taxa and a reduced relative abundance of certain putative opportunistic taxa; however, such taxonomic changes are correlative. and 16S-based data cannot verify viability or pathogenicity.

KEGG prediction further supports this functional optimization, showing an upregulation of microbial pathways for carbohydrate, amino acid, and energy metabolism. This suggests that OMS enhances the microbial metabolic potential, thereby improving nutrient utilization and energy harvest for the host. This establishes a virtuous cycle: an optimized microbiota structure enhances metabolic function, which in turn supports host health and performance. Recent work demonstrated that combining OEO with probiotics enhanced growth, immunity, antioxidant status, and microbiota balance in Eimeria-challenged broilers, supporting the broader potential of OEO-based combination strategies [47]. While both studies report improved immune and antioxidant outcomes, the mechanisms of synergy likely differ: probiotic-mediated modulation in their model versus complementary antimicrobial and anti-inflammatory effects in the OEO–MCE formulation. These distinctions suggest that different OEO-based combinations may enhance host physiology through different biological pathways.

## 5. Conclusions

In summary, this study demonstrates that the combined supplementation of OEO and MCE yielded greater improvements in broiler growth performance, serum biochemical profiles, antioxidant capacity, and immune indicators compared with either additive alone. While these findings suggest a potential synergistic benefit of the combined formulation, the underlying mechanisms cannot be inferred from the current data, and the associations between microbial shifts and host phenotypes should be considered correlational rather than causal. It is also important to acknowledge several limitations. First, the PICRUSt2-based functional profiling provides only predictive, inference-level insights rather than direct measurements of microbial metabolic activity. Second, intestinal histology was not evaluated, preventing direct confirmation of gut structural improvements. Third, metabolomic analyses were not performed, limiting our ability to validate microbial functional outputs and host–microbe metabolic interactions. Despite these limitations, the results collectively indicate that OEO and MCE may act in a complementary manner to support gut microbial balance, modulate systemic metabolic and immune responses, and enhance overall physiological performance. These findings highlight the strong potential of OMS as a safe and effective phytogenic strategy for sustainable, antibiotic-free poultry production, while underscoring the need for future mechanistic, histological, and metabolomic studies to validate the biological basis of the observed synergy.

## Figures and Tables

**Table 1:** Effects of OMS on broiler growth performance.

Items	Control	OEO	MCE	OMS	*p*-Value
ADFI (g)	56.08 ± 1.04 ^b^	56.99 ± 1.30 ^b^	56.94 ± 1.29 ^b^	59.06 ± 1.19 ^a^	0.003
ADG (g)	35.45 ± 1.44 ^c^	37.47 ± 1.22 ^b^	37.06 ± 1.31 ^b^	39.45 ± 1.28 ^a^	<0.001
F/G	1.48 ± 0.04 ^b^	1.46 ± 0.08 ^b^	1.53 ± 0.02 ^b^	1.64 ± 0.10 ^a^	0.001

^a, b, c^ Values within the same row with different superscript letters are significantly different at *p* < 0.05. Data are presented as Mean ± SD (*n* = 6). ADFI, average daily feed intake; ADG, average daily gain; F/G, feed-to-gain ratio.

**Table 2:** Effects of OMS supplementation on serum protein and lipid metabolic indices in broilers.

Items	Control	OEO	MCE	OMS	*p*-Value
TP (g/L)	28.49 ± 4.30 ^b^	30.64 ± 2.93 ^b^	29.42 ± 2.44 ^b^	35.65 ± 2.19 ^a^	0.003
ALB (g/L)	14.11 ± 1.31	13.81 ± 1.79	14.14 ± 1.46	14.12 ± 2.12	0.984
BUN (mmol/L)	1.19 ± 0.20 ^a^	0.86 ± 0.16 ^b^	0.88 ± 0.17 ^b^	0.75 ± 0.13 ^b^	0.001
TG (mmol/L)	0.88 ± 0.21 ^a^	0.90 ± 0.12 ^a^	0.88 ± 0.09 ^a^	0.61 ± 0.17 ^b^	0.011
TC (mmol/L)	4.26 ± 0.37 ^a^	3.91 ± 0.43 ^ab^	4.16 ± 0.20 ^a^	3.62 ± 0.23 ^b^	0.012
ALP (U/L)	1228.22 ± 16.23 ^a^	1233.88 ± 19.17 ^a^	1218.36 ± 23.14 ^ab^	1193.60 ± 32.31 ^b^	0.036

^a, b^ Values within the same row with different superscript letters are significantly different at *p* < 0.05. Data are presented as Mean ± SD (*n* = 6). TP, total protein; ALB, albumin; BUN, blood urea nitrogen; TG, triglycerides; TC, total cholesterol; ALP, alkaline phosphatase.

**Table 3:** Effects of OMS supplementation on serum antioxidant and immune indices in broilers.

Items	Control	OEO	MCE	OMS	*p*-Value
T-AOC (U/mL)	6.95 ± 0.31 ^c^	7.35 ± 0.20 ^ab^	7.17 ± 0.14 ^bc^	7.47 ± 0.20 ^a^	0.004
GSH-Px (U/mL)	258.96 ± 22.21 ^b^	288.21 ± 22.96 ^a^	290.84 ± 30.28 ^a^	312.65 ± 9.52 ^a^	0.005
MDA (U/mL)	6.39 ± 0.46	6.26 ± 0.26	6.36 ± 0.39	5.88 ± 0.70	0.261
SOD (U/mL)	175.75 ± 15.80 ^b^	187.57 ± 21.20 ^ab^	188.42 ± 12.46 ^ab^	205.47 ± 21.31 ^a^	0.070
IgA (U/mL)	204.86 ± 25.16 ^b^	245.93 ± 18.11 ^a^	242.14 ± 17.18 ^a^	264.87 ± 14.56 ^a^	<0.001
IgM (ng/mL)	9.69 ± 0.83 ^b^	11.20 ± 0.84 ^ab^	10.95 ± 1.59 ^ab^	11.72 ± 1.03 ^a^	0.032
IgG (ng/mL)	1.81 ± 0.16 ^b^	2.07 ± 0.25 ^ab^	2.20 ± 0.27 ^a^	2.19 ± 0.15 ^a^	0.017
TNF-α (pg/mL)	192.59 ± 13.83 ^a^	160.25 ± 16.44 ^bc^	169.39 ± 8.83 ^b^	152.83 ± 14.72 ^c^	<0.001
IL-1β (pg/mL)	88.36 ± 7.84 ^a^	69.67 ± 8.45 ^b^	72.23 ± 5.21 ^b^	65.34 ± 6.85 ^b^	<0.001
IL-4 (pg/mL)	58.84 ± 4.83 ^a^	45.23 ± 6.59 ^ab^	49.23 ± 8.14 ^b^	39.65 ± 5.67 ^c^	<0.001
IL-6 (pg/mL)	68.80 ± 12.28 ^a^	58.01 ± 16.25 ^ab^	62.84 ± 8.71 ^a^	43.09 ± 11.62 ^b^	0.013

^a, b, c^ Values within the same row with different superscript letters are significantly different at *p* < 0.05. Data are presented as Mean ± SD (*n* = 6). T-AOC, total antioxidant capacity; GSH-Px, glutathione peroxidase; MDA, malondialdehyde; SOD, superoxide dismutase; IgA, immunoglobulin A; IgM, immunoglobulin M; IgG, immunoglobulin G; TNF-α, tumor necrosis factor-α; IL-1β, interleukin-1β; IL-4, interleukin-4; IL-6, interleukin-6.

## Data Availability

The original contributions presented in this study are included in the article and Supplementary Materials. Further inquiries can be directed to the corresponding authors.

## Supplementary Materials

The following supporting information can be downloaded at https://www.mdpi.com/article/10.3390/vetsci12121206/s1, Table S1: Composition and nutrient levels of the basal diet (on air-dry basis).
