# Supplementary material for: Effects of Combined Oregano Essential Oil and Macleaya cordata Extract on Growth, Antioxidant Capacity, Immune Function, and Fecal Microbiota in Broilers
Source: Vet Sci. 2025 Dec 16;12(12):1206. doi: 10.3390/vetsci12121206 (PMC12737334; doi:10.3390/vetsci12121206)
Supplement: Supplementary file 1 [file vetsci-12-01206-s001.zip › Supplementary Materials.pdf]

Supplementary Table S1. Composition and nutrient levels of the basal diet (on air-dry basis)

Ingredients	Content (%)	Nutrient Levels	Calculated Value
Corn	60.00	Metabolic energy, MJ/kg	13.25
Soybean meal	28.40	Crude protein	21.00
Cottonseed meal	7.15	Crude fiber	7.00
Limestone	1.80	Crude ash	8.00
CaHPO4	1.60	Ca	1.00
NaCl	0.35	Total phosphorus	0.45
Lysine	0.35	Lysine	0.95
Vitamin premix [1]	0.20		
Trace element premix [2]	0.15		
Total	100.00		

[1] The vitamin premix provided the following per kg of diet: VA 5000 IU, VD_3 1000 IU, VE 10 IU, VB_2 3.6 mg, VB_{12} 0.01 mg.

[2] The trace element premix provided the following per kg of diet: Fe 60 mg, Cu 10 mg, Zn 45 mg, Mn 60 mg.
